# TGFβ Impairs HNF1α Functional Activity in Epithelial-to-Mesenchymal Transition Interfering With the Recruitment of CBP/p300 Acetyltransferases

**DOI:** 10.3389/fphar.2019.00942

**Published:** 2019-08-30

**Authors:** Francesca Bisceglia, Cecilia Battistelli, Valeria Noce, Claudia Montaldo, Agatino Zammataro, Raffaele Strippoli, Marco Tripodi, Laura Amicone, Alessandra Marchetti

**Affiliations:** ^1^Istituto Pasteur Italia–Fondazione Cenci Bolognetti, Department of Molecular Medicine, Sapienza University of Rome, Rome, Italy; ^2^National Institute for Infectious Diseases L. Spallanzani, IRCCS, Rome, Italy

**Keywords:** HNF1α, TGFβ, CBP/p300, histone acetylation, EMT, fibrosis, HCC

## Abstract

The cytokine transforming growth factor β (TGFβ) plays a crucial role in the induction of both epithelial-to-mesenchymal transition (EMT) program and fibro-cirrhotic process in the liver, where it contributes also to organ inflammation following several chronic injuries. All these pathological situations greatly increase the risk of hepatocellular carcinoma (HCC) and contribute to tumor progression. In particular, late-stage HCCs are characterized by constitutive activation of TGFβ pathway and by an EMT molecular signature leading to the acquisition of invasive and metastatic properties. In these pathological conditions, the cytokine has been shown to induce the transcriptional downregulation of HNF1α, a master regulator of the epithelial/hepatocyte differentiation and of the EMT reverse process, the mesenchymal-to-epithelial transition (MET). Therefore, the restoration of HNF1α expression/activity has been proposed as targeted therapeutic strategy for liver fibro-cirrhosis and late-stage HCCs. In this study, TGFβ is found to trigger an early functional inactivation of HNF1α during EMT process that anticipates the effects of the transcriptional downregulation of its own gene. Mechanistically, the cytokine, while not affecting the HNF1α DNA-binding capacity, impaired its ability to recruit CBP/p300 acetyltransferases on target gene promoters and, consequently, its transactivating function. The loss of HNF1α capacity to bind to CBP/p300 and HNF1α functional inactivation have been found to correlate with a change of its posttranslational modification profile. Collectively, the results obtained in this work unveil a new level of HNF1α functional inactivation by TGFβ and contribute to shed light on the early events triggering EMT in hepatocytes. Moreover, these data suggest that the use of HNF1α as anti-EMT tool in a TGFβ-containing microenvironment may require the design of new therapeutic strategies overcoming the TGFβ-induced HNF1α inactivation.

## Introduction

Transforming growth factor β (TGFβ) has emerged as a major microenvironmental factor playing a role in all phases of chronic liver diseases (Fabregat et al., 2016). This cytokine, in fact, is primarily involved in liver inflammation (by stimulating lymphocytes to produce inflammatory cytokines), in fibrosis (by activating the trans-differentiation of hepatic stellate cells to myofibroblasts and the subsequent production of large amount of extracellular matrix), and in the onset of hepatocellular carcinoma (HCC) that, in almost all the cases, develops on the described pathological tissue background (Amicone and Marchetti, 2018). Furthermore, once the tumor is established, the continuous production of TGFβ by both tumor and nontumor tissue, contributes to its growth and metastasization, mainly through the induction of epithelial-to-mesenchymal transition (EMT) in transformed hepatocytes (Zavadil and Bottinger, 2005). Accordingly, an unbalanced level of the cytokine in the tumor niche and high amount of the circulating cytokine have been shown to contribute to tumor progression and to a poor prognosis (Amicone et al., 2002; Lee et al., 2012).

TGFβ is a well-known inducer of EMT in several types of epithelial cells. In hepatocytes, the cytokine induces the trans-differentiation process through the upregulation of EMT/mesenchymal genes (Xu et al., 2009) and the strong transcriptional downregulation of master regulators of epithelial/hepatocyte differentiation, such as HNF4α and HNF1α (Marchetti et al., 2013).

In particular, TGFβ was shown to interfere with *HNF4α* and *HNF1α* gene expression in hepatocytes by upregulating the EMT master gene Snail, a transcriptional inhibitor that, in turn, induces HNF4α and HNF1α transcriptional repression through the direct binding to their promoters (Cicchini et al., 2006; Cozzolino et al., 2013; Battistelli et al., 2017).

HNF4α and HNF1α are well-known master regulators of hepatocyte differentiation, able to drive a complex epithelial/hepatocyte transcriptional program. Recently, it has been shown that, in fully differentiated hepatocytes, HNF4α and HNF1α are responsible not only for the maintenance of the epithelial program but also for a stable and continuous inhibition of the mesenchymal one, through the transcriptional repression of EMT/mesenchymal genes (Noce et al., in press; Santangelo et al., 2011). Furthermore, these proteins have been largely described as mesenchymal-to-epithelial transition (MET) master genes and tumor suppressors. HNF4α and HNF1α expression is lost during liver fibrosis and HCC progression (Lazarevich et al., 2004; Lazarevich et al., 2010; Willson et al., 2013; Ni et al., 2017), while their exogenous expression triggers growth arrest in hepatoma cell lines (Lazarevich et al., 2004; Pelletier et al., 2011) and induces hepatocyte differentiation in dedifferentiated cells (Santangelo et al., 2011). Most significantly, HNF4α and HNF1α delivery in animal models attenuates liver fibrosis (Yue et al., 2010; Song et al., 2016) and inhibits growth of xenograft tumors (Ning et al., 2010; Zeng et al., 2011).

For all these reasons, HNF4α and HNF1α have been proposed as therapeutic molecules for HCC (Marchetti et al., 2015).

However, recent data from our laboratory suggested that, in a TGFβ-containing environment, such as that in which HCC develops, the restoration of HNF4α function is not effective in suppressing the malignant behavior. We unveiled, in fact, a functional inactivation of HNF4α by TGFβ due to specific posttranslational modifications (PTMs) on the protein that correlate with the early loss of target gene promoters binding capacity (Cozzolino et al., 2013).

Here, we show that also HNF1α is subjected to a further level of TGFβ-induced downregulation, other than the transcriptional one. While TGFβ does not interfere with the HNF1α ability to bind to DNA, it negatively impairs HNF1α activity affecting its capacity to interact with CBP/p300 histone acetyltransferases. The loss of CBP/p300 recruitment on regulatory regions of HNF1α target genes, with consequent loss of a main transcription activating chromatin modification, prevents the HNF1α transcriptional function. Furthermore, we correlated the functional inactivation of HNF1α protein to a change in its PTM profile.

Altogether, our results demonstrate a new level of control of HNF1α by TGFβ that can represent the first event in triggering EMT process in hepatocyte and disclose a potential limitation to the use of an exogenous molecule as therapeutic MET inducer and tumor suppressor tool. However, and notably, the described mechanisms could allow the design of new therapeutic approaches aimed at overcoming the inactivating effect of the cytokine.

## Materials and Methods

### Cell Cultures and Treatments

Nontumorigenic murine hepatocytes (Amicone et al., 1997) and their Ras-transformed counterpart (Cozzolino et al., 2013) were grown on collagen-I-coated dishes in RPMI-1640 medium supplemented with 10% fetal bovine serum (GIBCO^®^ Life Technology, Monza, Italy), 50 ng/ml epidermal growth factor, 30 ng/ml insulin-like growth factor II (PeproTech Inc., Rocky Hill, NJ, USA), 10 µg/ml insulin (Roche, Mannheim, Germany), and antibiotics. Where indicated, cells were treated with 4 ng/ml of TGFβ1 (PeproTech Inc., Rocky Hill, NJ, USA) for the indicated time. As previously reported, cell lines utilized in this study undergo EMT following TGFβ treatment (Cozzolino et al., 2013; Grassi et al., 2015; De Santis Puzzonia et al., 2016; Battistelli et al., 2018).

HNF1α-overexpressing cells were obtained by transient transfection with pLPCX-HNF1α^Myc^ (carrying the rat HNF1α cDNA, Myc-tagged at the 5’ end). Control cell lines were obtained by transfection with the empty vector. Nontumorigenic and Ras-transformed hepatocytes were transfected with Lipofectamine 2000 (Invitrogen, San Diego, CA) or FuGENE^®^ HD Transfection Reagent (Promega Corporation, Madison, WI), respectively, according to the manufacturer’s protocol, and collected 48 h after transfection.

### RNA Extraction, Reverse Transcription, and Quantitative Real-Time PCR

Total RNAs were extracted with Total RNA Mini Kit (Geneaid) according to manufacturer’s protocol and reverse-transcribed using PrimeScript RT Master Mix (Takara, Dalian, China). cDNA was amplified by qPCR using GoTaq qPCR Master Mix (Promega Corporation, Madison, WI) in BioRad-iQ-iCycler. Relative amounts, calculated with the 2^(−ΔCt)^ method, were normalized with respect to the housekeeping gene RPL34 (60S ribosomal protein L34) or 18S rRNA. The sequence of primers utilized are listed in **Table 1**.

**Table 1:** List of mouse primers used for RT-qPCR experiments.

Gene	Forward primer	Reverse primer
*HNF4α*	5’-TCTTCTTTGATCCAGATGCC-3’	5’-GGTCGTTGATGTAATCCTCC-3’
*HNF1α*	5’-TATCATGGCCTCGCTACCTG-3’	5’ -ACTCCCCATGCTGTTGATGA-3’
*TTR*	5’-CCATGAATTCGCGGATGTGG-3’	5’-TCAATTCTGGGGGTTGCTGA-3’
*Albumin*	5’-TTCCTGGGCACGTTCTTGTA-3’	5’-GCAGCACTTTTCCAGAGTGG-3’
*18S*	5’-ACGACCCATTCGAACGTCTG-3’	5’ -GCACGGCGACTACCATCG-3’
*RPL34*	5’-GGAGCCCCATCCAGACTC-3’	5’ -CGCTGGATATGGCTTTCCTA -3’

### SDS-PAGE and Western Blotting

Cells were lysed in radioimmunoprecipitation assay buffer containing freshly added cocktail protease inhibitors [complete, ethylenediaminetetraacetic acid (EDTA)-free protease inhibitor cocktail; Sigma-Aldrich, St. Louis, MO]. Western Blots were performed as previously described (Cozzolino et al., 2013) using the following primary antibodies: rabbit polyclonal α-HNF1α (NBP1-33596, 1:1000; Novus Biologicals, USA), rabbit polyclonal α-CBP/p300 (451, 1:1000; Santa Cruz Biotechnology Inc., Santa Cruz, CA, USA), rabbit monoclonal α-cyclin-dependent kinase 4 (CD22, 1:1000; Santa Cruz Biotechnology Inc., Santa Cruz, CA, USA), and mouse monoclonal α-glyceraldehyde 3-phosphate dehydrogenase (MAB374, 1:1000; Millipore Corp., Bedford, MA, USA). Blots were then incubated with horseradish-peroxidase-conjugated species-specific secondary antibodies (Bio-Rad, Hercules, CA, USA), followed by enhanced chemiluminescence reaction (Bio-Rad Laboratories Inc., Hercules, CA, USA). Densitometric analyses were performed with ImageJ.

### Immunofluorescence Staining

For indirect immunofluorescence analysis, cells were fixed in 4% paraformaldehyde, permeabilized with 0.2% Triton-X100, and incubated with α-HNF1α antibody (NBP1-33596, 1:50; Novus Biologicals, USA), α-Myc-Tag antibody (9B11, 1:200; Cell Signaling Technologies Inc. Danvers, USA), and E-cadherin antibody (610182, 1:50; BD Biosciences). Alexa Fluor 488-conjugated and Alexa Fluor-594-conjugated secondary antibodies (1:400; Molecular Probes, Eugene, OR, USA) were utilized. Nuclei were stained with 4’,6-diamidino-2-phenylindole (DAPI; Calbiochem Merck, Darmstadt, Germany). Images were examined with a Nikon Eclipse microscope (Nikon Corporation, Tokyo, Japan) equipped with a charge-coupled device camera. Digital images were acquired by Nikon NIS elements software (Nikon Corporation, Tokyo, Japan) and processed with Adobe Photoshop 7 software (Adobe Systems, Mountain View, CA). The same enhanced color levels were applied for all channels.

### Chromatin Immunoprecipitation

Chromatin immunoprecipitation (ChIP) analysis was performed as previously reported (Cozzolino et al., 2016; Battistelli et al., 2017) using 5 μg of the following antibodies for the immunoprecipitation: goat polyclonal α-HNF1α (C-19; Santa Cruz Biotechnology Inc., Santa Cruz, CA, USA), rabbit polyclonal α-CBP (451; Santa Cruz Biotechnology Inc., Santa Cruz, CA, USA), rabbit polyclonal α-acetyl H3 (06-599; Millipore Corp., Bedford, MA, USA), normal rabbit antiserum (Millipore Corp., Bedford, MA, USA), and normal goat IgG (AB-108-C; R&D Systems, Minneapolis, USA) were used as negative controls. Equal amounts of immunoprecipitated DNA and relative controls were used for qPCR analysis, performed in triplicate. The list of primers utilized is shown in **Table 2**. qPCR analysis of the immunoprecipitated samples and of the negative controls (IgG) were both normalized to total chromatin input. The promoter of *Neurogenin 1*, a gene not expressed in hepatocytes, was used as negative control.

**Table 2:** List of mouse primers used for qPCR in ChIP experiments.

Promoter	Forward primer	Reverse primer
*Albumin*	5’-AGGAACCAATGAAATGCGAGG-3’	5’-AGACGAAGAGGAGGAGGAGA-3’
*HNF4α*	5’-ACTTGGGCTCCATAGCAAGA-3’	5’- CAGGACAGGCACAGACAAGA-3’
*Neurog1*	5’-CCTCCCGCGAGCATAAATTA-3’	5’-GCGATCAGATCAGCTCCTGT -3’
*RPL30*	5’-TAAGGCAGGAAGATGGTGG -3’	5’- CAGTGTGCTCAAATCTATCC-3’

### Electrophoretic Mobility Shift Assay

Cells were scraped in cold phosphate-buffered saline (PBS), lysed in 10 mM Hepes pH 7.9, 1.5 mM MgCl_2_, 10 mM KCl, 0.1% NP40, 0.1 mM EDTA, 0.5 mM dithiothreitol (DTT), standard protease and phosphatase inhibitors, and centrifuged to pellet the nuclei. Nuclear proteins were extracted in 20 mM Hepes pH 7.9, 20% glycerol, 0.42 M NaCl, 1.5 mM MgCl_2_, 0.2 mM EDTA, 0.1% NP40, 0.5 mM DTT, and standard protease inhibitors. Protein concentrations were determined with the Bio-Rad Protein Assay Dye Reagent (Bio-Rad Laboratories, Hercules, CA).

For nonradioactive electrophoretic mobility shift assay (EMSA), biotin end-labeled oligonucleotide probes were obtained with the Biotin 3′ End DNA Labeling Kit (Thermo Fisher Scientific, Waltham, MA, USA), according to manufacturer’s protocol. The sequences of oligonucleotides used are the followings: for the mouse HNF4α promoter, 5’-CGGGGTGATTAACCATTAACTCCTACCCCT-3’ and 5’-AGGGGTAGGAGTTAATGGTTAATCACCCCG-3’ (the HNF1α binding site is underlined); for the mouse ApoC3 promoter, 5’-CAGCAGGTGACCTTTGCCCAGCTCAC-3’ and 5’-GTGAGCTGGGCAAAGGTCACCTGCTG-3’ (the HNF4α binding site is underlined).

Gel shift assays were performed using LightShift Chemiluminescent EMSA Kit (Thermo Fisher Scientific, Waltham, MA, USA), according to the manufacturer’s protocol. The binding reaction was prepared in a final volume of 20 µl incubating 1× binding buffer, 2.5% glycerol, 5 mM MgCl_2_, 50 ng/µl PolydI-dC, 0.05% NP-40, and 10 µg of nuclear extracts (except for the free probe sample) for 10’ at 4°C. Then, 25 fmol of the double-strand biotinylated probe were added and the reaction conducted for further 20’ at RT. Where specified, a 100-fold excess of unlabeled annealed oligonucleotide or 5 µg of the following antibodies were added to the binding reaction before addition of nuclear extracts: rabbit polyclonal α-HNF1α (H-140; Santa Cruz Biotechnology Inc., Santa Cruz, CA, USA) or rabbit polyclonal α-HNF1α (NBP1-33596; Novus Biologicals) and mouse monoclonal α-tubulin (TU-02; Santa Cruz Biotechnology Inc., Santa Cruz, CA, USA). Samples were loaded on a 6% nondenaturing polyacrylamide gel in 0.5× tris borate EDTA and transferred to a nylon membrane (Biodyne B Nylon Membrane, Thermo Fisher Scientific, Waltham, MA, USA). After cross-linking to the membrane at 120 mJ/cm^2^ for 1’ with UV Stratalinker 1800 (Stratagene, San Diego, CA, USA), biotin-labeled DNA was detected using Chemiluminescent Nucleic Acid Detection Module Kit (Thermo Fisher Scientific, Waltham, MA, USA).

### Co-immunoprecipitation

Cells were lysed in IP lysis buffer (150 mM NaCl, 50 mM Tris–HCl pH 7.5, 2 mM EDTA, 1% Triton-X100, 10% glycerol) supplemented with protease and phosphatase inhibitors. One milligram of cell lysates, after preclearing with protein A-Sepharose (GE Healthcare, Little Chalfont, Buckinghamshire, UK), was incubated with 5 µg of goat polyclonal α-HNF1α antibody (C-19; Santa Cruz Biotechnology Inc., Santa Cruz, CA, USA) or normal goat immunoglobulin G (IgG) (AB-108-C; R&D Systems, Minneapolis, USA) at 4°C overnight while rotating. Immunocomplexes were then incubated on a rotating platform for 3 h with protein A-sepharose at 4°C, washed in NetGel buffer (150 mM NaCl; 50 mM Tris–HCl pH 7.5; 1 mM EDTA; 0.1% NP-40; 0.25% gelatin), eluted and denatured in Laemmli buffer. Proteins from immunoprecipitation were resolved on sodium dodecyl sulfate polyacrylamide gel electrophoresis (SDS-PAGE) and transferred to nitrocellulose membrane (Bio-Rad Laboratories, Hercules, CA). For immunoblotting, the following primary antibodies were used: rabbit polyclonal α-HNF1α (NBP1-33596, 1:2000; Novus Biologicals) and rabbit polyclonal α-CBP (451, 1:1000; Santa Cruz Biotechnology, Santa Cruz, CA). Immune complexes were detected with horseradish peroxidase-conjugated species-specific secondary antiserum (Bio-Rad Laboratories, Hercules, CA), followed by enhanced chemiluminescence reaction (Bio-Rad Laboratories Inc., Hercules, CA, USA).

### Two-Dimensional Gel Electrophoresis

Two-dimensional gel electrophoresis (2-DE) was performed using IPGphor II (GE Healthcare) as previously described (Cozzolino et al., 2013). In brief, proteins (90 µg) from nuclear extracts were precipitated with 100% acetone and then loaded on pH 3–10 IPG strips (IPGs) and electrofocused at 15,000 V/h at a maximum voltage of 5,000 V. The second-dimension separation was performed at a constant current of 50 mA for 2 h. Proteins were transferred to nitrocellulose membranes (Protran Nitrocellulose Transfer Membrane, Schleicher & Schuell, BD Biosciences), and Western blot was performed as described above with mouse monoclonal anti-Myc-Tag antibody (9B11, 1:1,000; Cell Signaling Technologies Inc. Danvers, USA).

### Statistical Analysis

Statistical significance was determined using paired one-tailed Student’s *t*-test or one-sample Student’s *t*-test. A *p* < 0.05 was considered statistically significant (*p < 0.05; **p < 0.01; ***p < 0.001).

## Results

### TGFβ Early Impairs HNF1α Functional Activity

It has previously reported that TGFβ is able to interfere with the activity of the hepatocyte differentiation master gene HNF4α, negatively controlling both gene expression and protein function (Santangelo et al., 2011; Cozzolino et al., 2013; Battistelli et al., 2017). These findings had seriously questioned the possibility of using HNF4α as therapeutic molecule. In order to verify the possibility to use alternatively HNF1α as MET inducer in a TGFβ-rich microenvironment, we explored the effect of this cytokine on the HNF1α protein function.

To this aim, we utilized liver cell lines, already described in our laboratory, as models of hepatocytes at different stages of differentiation (Amicone et al., 1997; Bellovino et al., 1998; Grassi et al., 2015) and able to undergo EMT upon TGFβ treatment (Grassi et al., 2015; De Santis Puzzonia et al., 2016). The first evidence of an additional level of HNF1α negative control induced by TGFβ came from a time course analysis of HNF1α-dependent gene expression regulation in hepatocytes treated with the cytokine.

**Figure 1:**
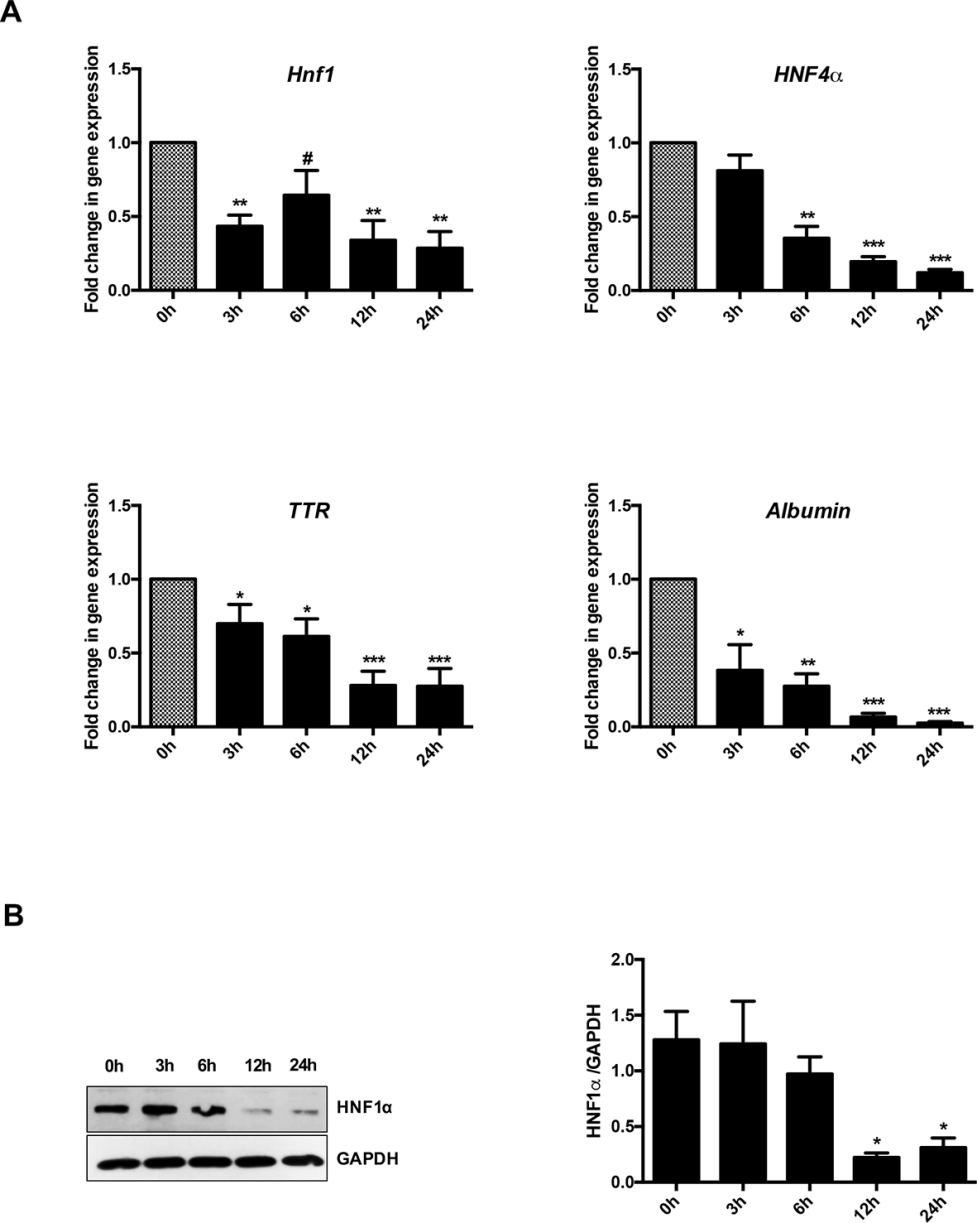
Analysis of HNF1α transcriptional activity in hepatocytes following transforming growth factor β (TGFβ) treatment. **(A)** Gene expression analysis by RT-qPCR of HNF1α and the indicated target genes in hepatocytes in a time-course experiment following TGFβ treatment. qPCR data, obtained in triplicate and normalized to the ribosomal RNA 18S, are expressed as fold change in gene expression in TGFβ-treated versus untreated cells (arbitrary value = 1) (^#^p <0.06). The mean ± SEM of three independent experiments is shown. The observed differences in gene expression are statistically significant (*p < 0.05; **p < 0.01; ***p < 0.001). **(B)** Western blot analysis for HNF1α in protein extracts from one of the experiments shown in **(A)**. α-Glyceraldehyde 3-phosphate dehydrogenase was used as loading control. Densitometric analysis of WB data from three independent experiments is shown.

**Figure 1A** shows that, as expected, TGFβ induced a significant and early transcriptional downregulation of HNF1α (at 3 h of treatment) and of its target genes *HNF4α* (at 6 h of treatment), *Albumin* and *Transthyretin* (TTR) (at 3 h of treatment). Notably, the downregulation of the target genes occurred when the reduction in the HNF1α protein was not yet observable (**Figure 1B**), thus suggesting a functional inactivation of HNF1α protein by TGFβ that precedes the effects of the transcriptional downregulation of its own gene.

To formally prove the posttranslational control of HNF1α by TGFβ, we constitutively expressed an exogenous HNF1α in a not fully differentiated hepatocyte cell line and in Ras-transformed hepatocytes (Cozzolino et al., 2013).

**Figure 2:**
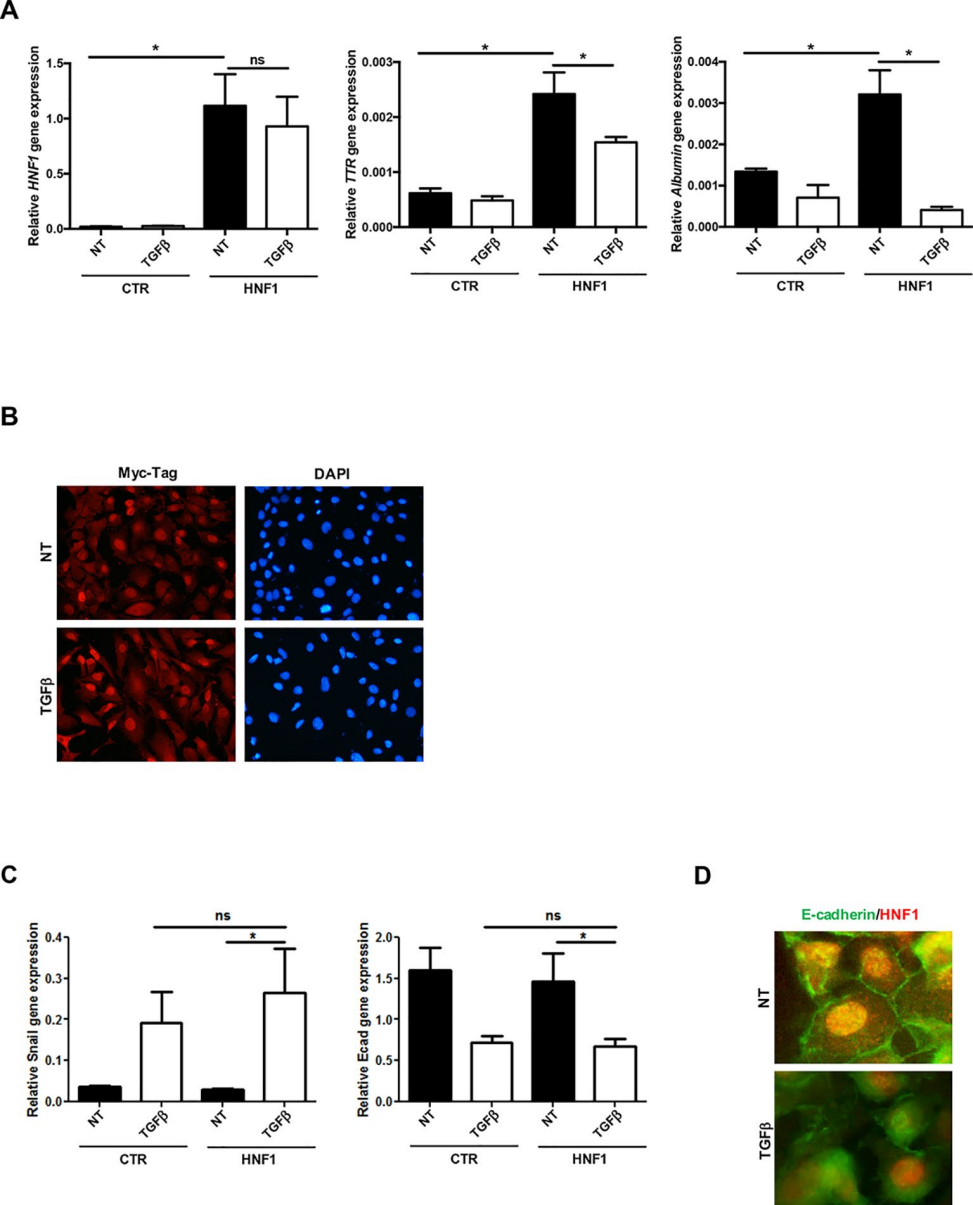
TGFβ overrides HNF1α constitutive expression. **(A)** Analysis of constitutively expressed HNF1α activity following TGFβ treatment. RT-qPCR analysis for the indicated genes in hepatocytes, transiently transfected with pLCPX-HNF1α^Myc^ (HNF1) or the empty vector (CTR), treated with 4 ng/ml TGFβ for 24 h or left untreated (NT). qPCR data, obtained in triplicate and normalized to the housekeeping gene RPL34, are expressed as relative gene expression. The mean ± SEM of three independent experiments is shown. Statistically significant differences are reported (*p < 0.05; ns, not significant). **(B)** Immunofluorescence analysis of hepatocytes transfected and treated as in **(A)**. Cells were stained with anti-MycTag antibody (red) and 4’,6-diamidino-2-phenylindole (nuclei, blue). Magnification 10×. **(C)** Analysis of EMT-related gene expression by RT-qPCR in hepatocytes, transiently transfected with pLCPX-HNF1αMyc (HNF1) or the empty vector (CTR), treated with TGFβ for 24 h or left untreated (NT). qPCR data, obtained in triplicate and normalized to the housekeeping gene RPL34, are expressed as relative gene expression. The mean ± SEM of three independent experiments is shown. Statistically significant differences are reported (*p < 0.05; ns, not significant). **(D)** Immunofluorescence analysis of hepatocytes transfected and treated as in **(C)**. Cells were stained with anti-E-cadherin (green) or anti-HNF1α antibody (red). Magnification 20×.

HNF1α target gene expression (*Albumin* and *TTR*), markedly induced by the exogenous HNF1α, was significantly reduced by TGFβ in both cell lines (**Figure 2A** and **Supplementary Figure S1A**), while the ectopic HNF1α protein level (**Supplementary Figure S1B**) and its nuclear localization (**Figure 2B**) were not modified. Furthermore, exogenous HNF1α was not able to hamper the TGFβ-induced EMT, as indicated by the transcriptional upregulation of Snail (**Figure 2C**), the downregulation and delocalization of E-cadherin (**Figures 2C, D**), and by the morphological transition (**Figure 2B**).

These data unveiled the dominance of TGFβ on HNF1α overexpression, both in the regulation of target gene expression and in the induction of EMT, thus confirming its ability to negatively control HNF1α at posttranscriptional level.

### TGFβ Does Not Affect DNA Binding Capacity of HNF1α

**Figure 3:**
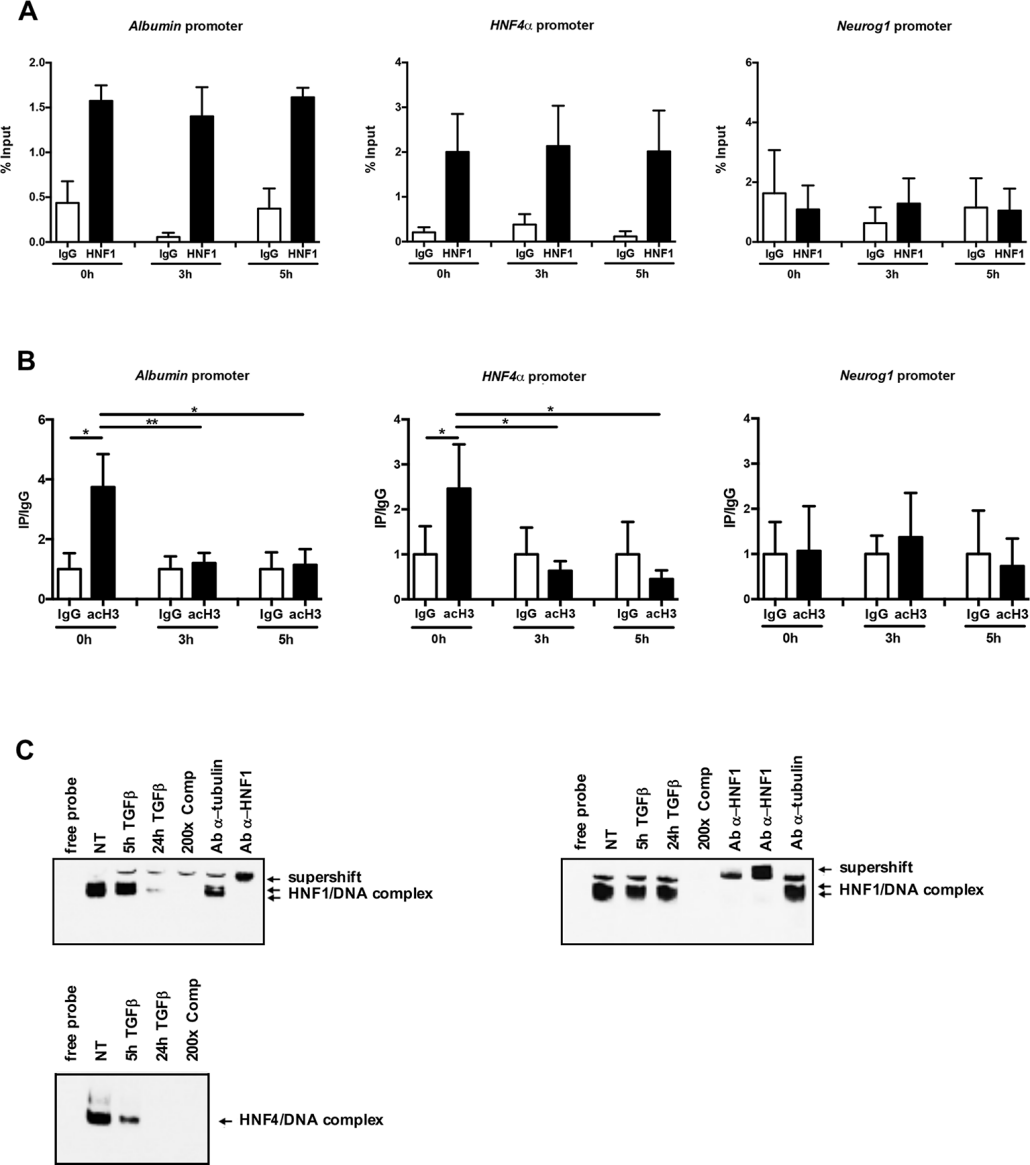
**(A)** HNF1α DNA binding activity after TGFβ treatment. qPCR analysis of chromatin immunoprecipitated from hepatocytes with anti-HNF1α antibody was performed. HNF1α consensus regions embedded in the indicated HNF1α target gene promoters were analyzed. A HNF1α nonbound region of *Neurogenin 1* promoter was utilized as negative control. Data are normalized to total chromatin input and background (control immunoprecipitation with IgG) and expressed as % input. Mean ± SEM of qPCR data obtained in triplicate from three independent experiments are reported. **(B)** Analysis of acetyl histone H3 by chromatin immunoprecipitation assay in untreated and TGFβ-treated hepatocytes. qPCR analysis of chromatin immunoprecipitated from hepatocytes with anti-acetyl H3 antibody was performed. HNF1α consensus regions embedded in the indicated HNF1α target gene promoters were analyzed. A HNF1α nonbound region of *Neurogenin 1* promoter was utilized as negative control. Data are normalized to total chromatin input and background (control immunoprecipitation with IgG) and expressed as (Ip/IgG) % input. The mean ± SEM of qPCR data obtained in triplicate from five independent experiments are reported. *p <0.05, **p <0.01. **(C)** Electrophoretic mobility shift assay (EMSA) assays. Nuclear extracts from untreated (NT) or TGFβ-treated (for 5 or 24 h) parental (left, upper panel) or HNF1α-overexpressing hepatocytes (right, upper panel) were analyzed for the binding to HNF1α consensus site within the murine HNF4α promoter. The presence of the HNF1α protein in the protein/DNA complexes was revealed by the band supershift obtained with the addition of two different anti-HNF1α antibodies. A 200-fold excess of unlabeled oligonucleotide and the antitubulin antibody were added to the untreated extracts to test the binding specificity. As control, HNF4α DNA binding activity to its consensus site within the ApoC3 promoter was analyzed by EMSA in the same extracts (lower panel).

In an attempt to investigate the mechanisms involved in TGFβ-dependent HNF1α inactivation, HNF1α binding to regulatory sequences of its target genes *HNF4α* and *Albumin* has been evaluated by a chromatin immunoprecipitation (ChIP) assay, at early time of TGFβ treatment. As shown in **Figure 3A**, the HNF1α DNA binding activity was not affected by TGFβ even after 5 h of treatment. Later time points were not analyzed since endogenous HNF1α was transcriptionally downregulated upon TGFβ treatment, as reported above.

However, and coherently with transcriptional data, the chromatin regions around the HNF1α binding site showed, at early time points after TGFβ treatment, a significant downregulation of the histone H3 acetylation, one of the main transcriptional activating chromatin modifications (**Figure 3B**).

Further evidence of the maintenance of HNF1α binding capacity in TGFβ-treated cells have been obtained by EMSA experiments. As shown in **Figure 3C**, the HNF1α/DNA complexes were observed and maintained until 5 h of TGFβ treatment (when the endogenous protein is still expressed, as shown in **Figure 1B**) in untreated parental hepatocytes and until 24 h of treatment in hepatocytes constitutively expressing HNF1α, thus confirming that the mechanism involved in the HNF1α inactivation does not impact on its DNA binding ability. On the contrary, and as expected, in the same extracts, the binding of endogenous HNF4α on its own consensus site within the promoter of *ApoC3* gene, was lost at early time points (5 h) of TGFβ treatment (**Figure 3C**, lower panel).

Overall, these data showed that TGFβ-induced functional inactivation of HNF1α does not depend on the loss of its DNA binding, but rather to the impairment of its ability to drive transcription activating chromatin modifications.

### TGFβ Induces HNF1α Functional Inactivation Interfering With the Recruitment of CBP/p300 Acetyltransferases

**Figure 4:**
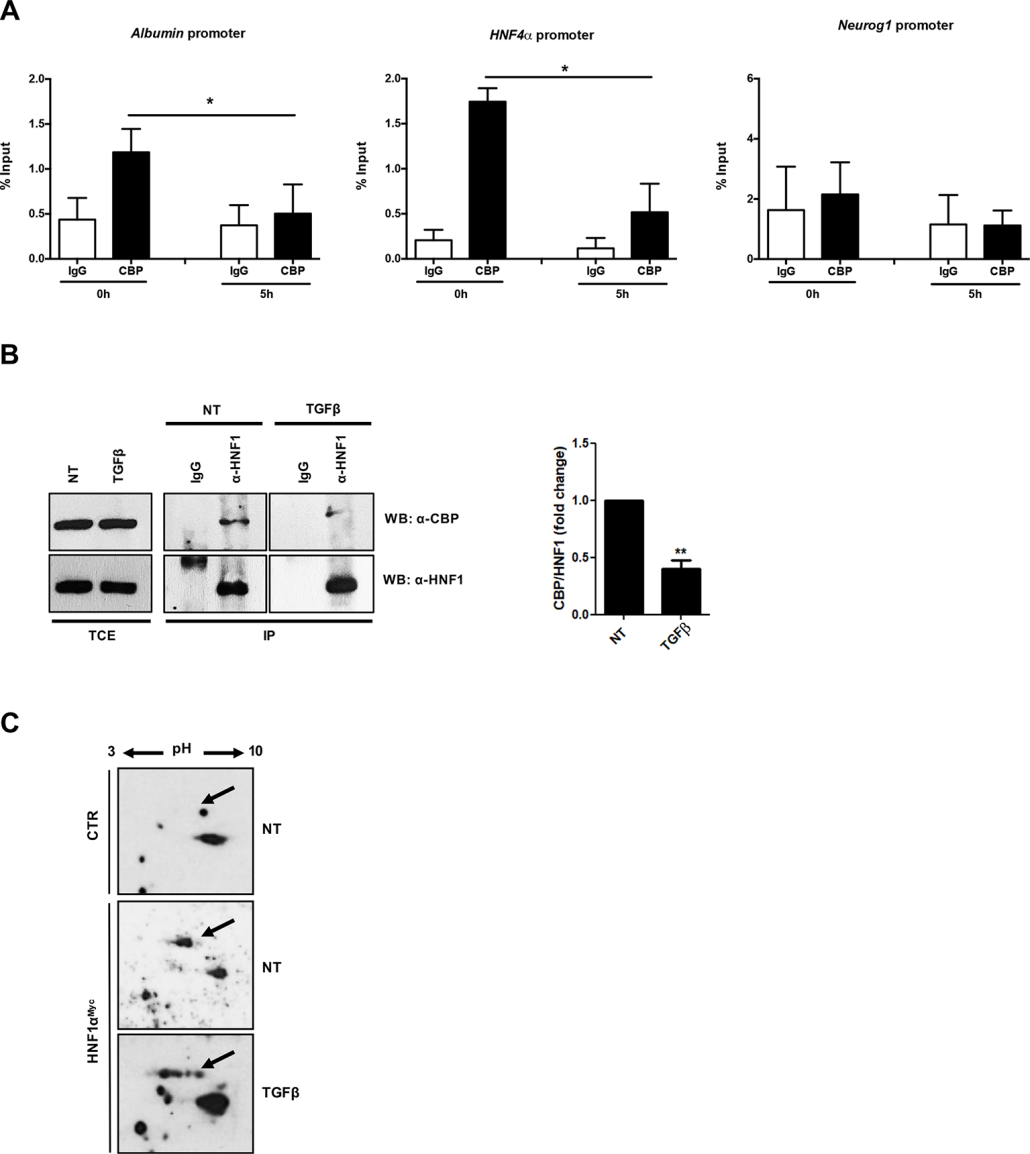
**(A)** Analysis of CBP/p300 DNA binding activity after TGFβ treatment. qPCR analysis of chromatin immunoprecipitated with anti-CBP/p300 antibody from hepatocytes untreated or treated with TGFβ (for 5 h) was performed. HNF1α consensus regions embedded in the indicated target gene promoters were analyzed. A HNF1α nonbound region of *Neurogenin 1* promoter was utilized as negative control. Data are normalized to total chromatin input and background (control immunoprecipitation with IgG) and expressed as % input. Mean ± SEM of qPCR data obtained in triplicate from three independent experiments are reported. **(B)** *In vivo* coimmunoprecipitation of HNF1α and CBP/p300 in hepatocytes untreated or treated with TGFβ (for 5 h). Cells were lysed, immunoprecipitated with anti-HNF1α antibody, and then analyzed by Western blotting with the indicated antibodies. As control, the immunoprecipitation with normal goat IgG was performed. TCE, total cell extracts. Densitometric analysis of WB data from three independent experiments is shown. **(C)** Analysis of HNF1α protein PTMs following TGFβ treatment. Nuclear extracts from control hepatocytes (upper panel) and HNF1α^Myc^-overexpressing hepatocytes treated for 3 h with TGFβ (lower panel) or left untreated (middle panel). Samples were separated by two-dimensional gel electrophoresis followed by Western blotting with anti-Myc-Tag antibody. HNF1α-specific spots are indicated by the arrow. The appearance of multiple spots in TGFβ-treated hepatocytes can be observed. **p <0.05, **p<0.01.

The correlation between the TGFβ-induced HNF1α functional inactivation and the loss of histone acetylation at its specific binding sites prompted us to investigate on the possible interference of TGFβ with the recruitment of histone acetyltransferase on the HNF1α target gene promoters. It has been previously shown that HNF1α interacts with the histone acetyltransferases CBP/p300 on target gene promoters (Ban et al., 2002; Dohda et al., 2004) and that the HNF1α-dependent nucleosome hyperacetylation is required for the activation of tissue-specific target genes (Parrizas et al., 2001). Thus, we analyzed by ChIP the effects of the TGFβ treatment on the CBP/p300 occupancy of HNF1α binding sites embedded in *Albumin* and *HNF4α* gene promoters. Our results demonstrated the presence of CBP/p300 in the untreated sample and, interestingly, the early displacement of these proteins upon TGFβ treatment (**Figure 4A**).

To investigate on the mechanism involved in the lack of histone acetyltransferase recruitment on DNA, the physical interaction between CBP/p300 and HNF1α has been evaluated in a coimmunoprecipitation assay, in the absence or in the presence of TGFβ (at 5 h of treatment). As shown in **Figure 4B**, the antibody specifically recognizing HNF1α was able to immunoprecipitate CBP/p300 acetyltransferase in untreated hepatocytes, while the TGFβ treatment early reduces this protein–protein interaction. Notably, the total amount of CBP/p300 was not affected by TGFβ treatment.

Overall, these findings indicate that the loss of physical interaction between of CBP/p300 and HNF1α, with consequent displacement of the acetyl-transferase activity from the regulatory regions of HNF1α target genes, represents the first step of TGFβ-induced HNF1α inactivation, contributing to the onset of EMT in hepatocytes.

### TGFβ Induces PTMs of HNF1α Protein

Our previous report unveiled that TGFβ induces a modification of HNF4α phosphorylation profile responsible for protein functional inactivation (Cozzolino et al., 2013). Thus, in the attempt to further investigate on the mechanism responsible for the reduced interaction between HNF1α and CBP/p300, we analyzed the effect of TGFβ on HNF1α PTM profile. To this aim, we performed a two-dimensional gel electrophoresis with nuclear extracts from untreated and TGFβ-treated hepatocytes (3 h), ectopically expressing HNF1α^Myc^, followed by Western blot with a Myc-Tag-specific antibody. As shown in **Figure 4C**, TGFβ strongly affects the PTM pattern on HNF1α protein. In particular, new specific “spot trains,” compatible with multiple phosphorylation/dephosphorylation events, and probably revealing intermediate isoforms, were observed. Some of these modifications might account for the observed altered interaction of HNF1α with CBP/p300, even if we cannot exclude the presence of additional PTMs nor the involvement of additional mechanisms (e.g., modulation of cofactors induced by TGFβ) that can affect protein complex formation.

## Discussion

The major contribution of the present work has been to unveil a novel mechanism by which TGFβ early affects the trans-activating function of the MET master gene HNF1α in triggering EMT process in hepatocytes.

The pleiotropic TGFβ cytokine has emerged as a pivotal player in hepatocarcinogenesis, taking part in the interplay between microenvironment and liver cells from initial liver injury and inflammation through fibro/cirrhosis to tumor onset, growth, and metastasization (Amicone and Marchetti, 2018). In particular, at late stage of hepatocarcinogenesis, the unbalanced level of the cytokine in the tumor niche can drive transformed hepatocytes towards an EMT and, ultimately, to the acquisition of migratory and invasive properties (Fabregat et al., 2016). Accordingly, in HCC patients, it has been observed that the constitutive activation of TGFβ signaling contributes to tumor progression and is associated with a poor prognosis (Lee et al., 2012).

One of the key events during the progression of hepatocellular carcinoma is the loss of expression of master genes of epithelial/hepatocyte differentiation, such as *HNF4α* and *HNF1α*, that play a pivotal role in the restraint of inflammation, fibrosis, and EMT (Yue et al., 2010; Hatziapostolou et al., 2011; Pelletier et al., 2011; Santangelo et al., 2011; Qian et al., 2015).

Our previous data demonstrated the ability of TGFβ signaling to downregulate *HNF4α* and *HNF1α* gene expression through the Snail-mediated transcriptional repression and, even before the transcriptional control, to affect the HNF4α activity by inhibiting its DNA binding capacity (Cozzolino et al., 2013). Results of this work demonstrated that TGFβ is able to early inactivate also HNF1α, acting at posttranslational level.

These observations suggest that the use of HNF1α, elsewhere proposed as therapeutic tool in the control of liver fibrosis and tumor development, could be ineffective in an *in vivo* TGFβ-containing microenvironment. On the other hand, the knowledge of the molecular basis of the TGFβ-induced HNF1α inactivation results is necessary to design new therapeutic approaches based on the use of molecules resistant to the inactivating effect of the cytokine.

Here, we demonstrated that (i) the functional inactivation of HNF1α protein by TGFβ precedes the effects of the transcriptional downregulation of its gene, (ii) TGFβ does not interfere with the HNF1α DNA-binding capability or its subcellular localization but induces a local reduction of chromatin acetylation at the HNF1α binding sites within target gene promoters, and (iii) TGFβ interferes with the recruitment of CBP/p300 acetyltransferases by HNF1α on target gene promoters, which could be due to a change of PTMs on HNF1α protein.

To accomplish its functions on specific targets, HNF1α often cooperates with coactivators or corepressor, including CBP/p300 acetyltransferases that play an important role in positively regulating transcription of hepatocyte-specific genes.

In general, p300 and CBP seem to act as transcriptional coactivators by bridging the activators to the basal transcriptional machinery and, through their histone acetyltransferase (HAT) activity, by modifying chromatin structure to a locally open and transcriptionally active configuration (Chan and La Thangue, 2001).

HNF1α/CBP and HNF1α/p300 physical interactions have been previously reported (Soutoglou et al., 2000; Ban et al., 2002; Dohda et al., 2004). Furthermore, both CBP and p300 were found to interact with HNF1α on the Albumin promoter and to cooperatively enhance its expression in primary hepatocytes (Dohda et al., 2004). The ability of HNF1α to direct nucleosome hyper-acetylation to target genes is fundamental for its transcriptional activity. A study carried out with hnf1α−/− mice models demonstrated that the organ-specific induction of different targets is strongly dependent on nucleosome acetylation (Parrizas et al., 2001). Our results confirmed the pivotal role of CBP/p300 as transcriptional coactivator of HNF1α and indicated the impairment of the interaction between the two proteins as an effective and early mechanism utilized by TGFβ to neutralize the HNF1α activity in the first phase of EMT process.

It has been described for some transcriptional factors known to recruit CBP and p300 on target genes the role of specific PTMs in mediating the physical protein–protein interaction (Chrivia et al., 1993; Wang et al., 2013). Previous proteomic studies on liver cells highlighted the presence of PTMs on HNF1α protein. However, at present, there are only a few studies showing the role of these PTMs on its functional activity (Lim et al., 2002; Zhao et al., 2014; Kaci et al., 2018). Data obtained by 2-DE gel electrophoresis analysis suggest that the regulation of HNF1α-CBP/p300 interaction by TGFβ could include HNF1α posttranslational modifications. Further proteomic analysis will allow the identification of residues involved and their functional significance. We cannot exclude, in fact, that additional mechanisms could contribute to the impairment of HNF1α activity by TGFβ.

The early neutralization of HNF1α activity has great relevance in the induction of EMT process in hepatocyte. In differentiated hepatocyte, in fact, it has been shown that HNF1α, as well as HNF4α, stably inhibits the expression of the EMT master gene Snail and, consequently, the mesenchymal program and that this control is mandatory for the maintenance of the differentiated phenotype (Santangelo et al., 2011; Battistelli et al., 2018). The late events involved in the TGFβ-dependent downregulation of both HNF1α and HNF4α have been previously characterized. Our previous works, indeed, described their transcriptional downregulation by TGFβ through the recruitment of Snail to HNF1α and HNF4α promoters with subsequent chromatin remodeling and transcriptional repression (Santangelo et al., 2011; Battistelli et al., 2017).

**Figure 5:**
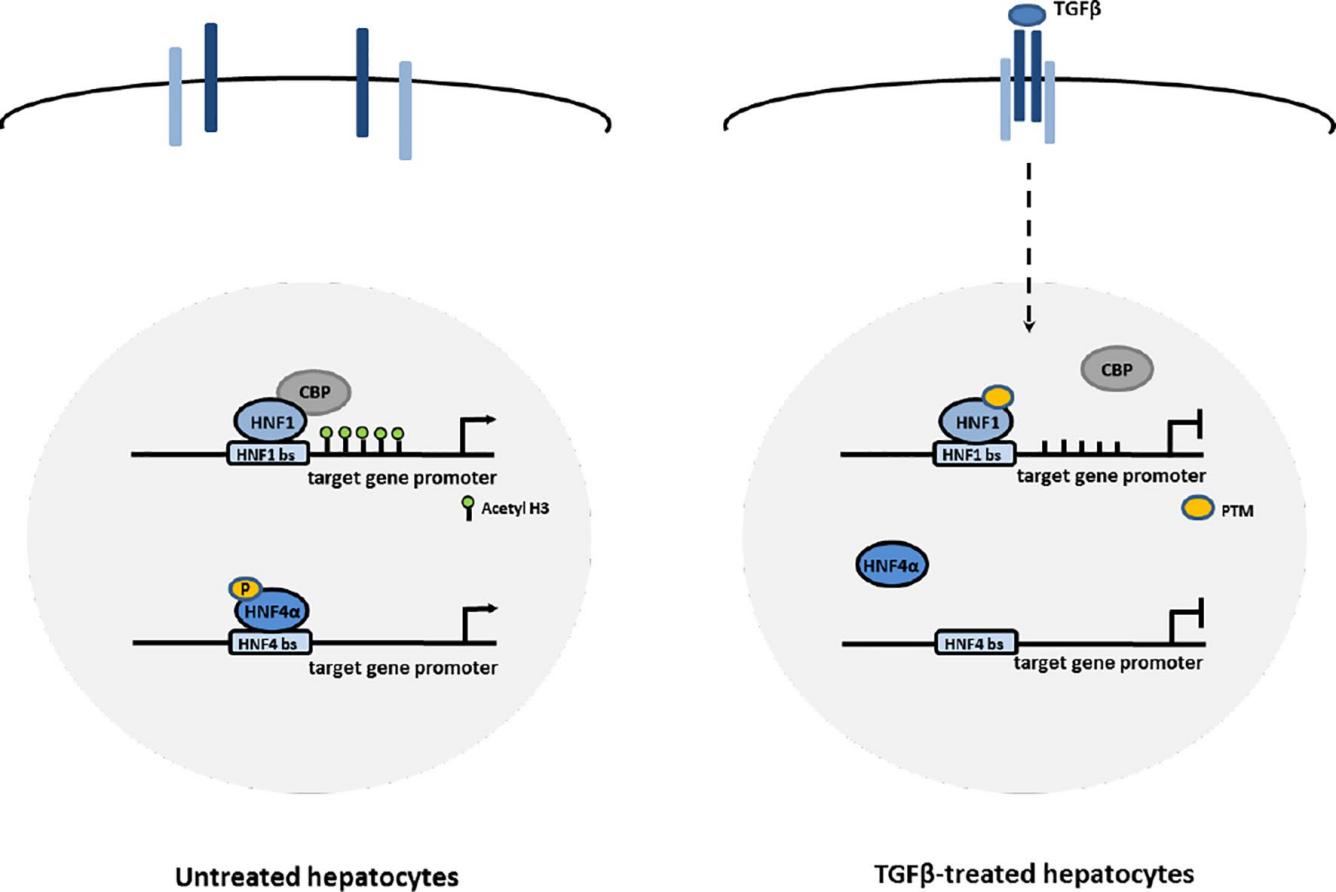
Proposed mechanisms of HNF1α and HNF4α inactivation by TGFβ in EMT. In hepatocytes, TGFβ induces the impairment of HNF4α DNA binding ability through the loss of activating phosphorylations of the protein and the impairment of HNF1α functional activity through the displacement of CBP/p300 acetyltransferases from target gene promoters, possibly mediated by change in PTM profile.

Thus, the early functional inhibition of HNF1α and HNF4α by TGFβ (even if through different mechanisms, as shown in **Figure 5**) could represent the first mechanism through which the expression of Snail and the mesenchymal program are released, and the EMT process is triggered.

In conclusion, the data presented here shed light on an early mechanism inhibiting HNF1α function during the first step of EMT process in hepatocytes and, consequently, unveils a potential limitation of the use of HNF1α as therapeutic tool for anti-EMT and antifibrosis molecular therapies in an *in vivo* TGFβ-containing microenvironment. Further characterization of the change in the HNF1α PTM profile, induced by TGFβ, could allow the design of new therapeutic approaches (i.e., mutant molecules resistant to the induction of these modifications) aimed to override the inactivating effect of the cytokine.

## Data Availability

The raw data supporting the conclusions of this manuscript will be made available by the authors, without undue reservation, to any qualified researcher.

## Author Contributions

FB, MT, and AM contributed to the design of the research plan and to the interpretation of results. FB, CB, VN, CM, and AZ performed the experiments. CB and VN contributed to the analysis and representation of data. AM and LA wrote the manuscript. MT and RS contributed to the critical revision of the manuscript. AM, LA, and MT revised the final draft of the manuscript. AM coordinated the experimental work. Financial support: MT, LA, AM.

## Funding

This study was supported by Sapienza University of Rome (Progetti di Ateneo: C26A15CL7B and RM118143646188C and by Associazione Italiana per la Ricerca sul Cancro (AIRC, IG 18843).

## Conflict of Interest Statement

The authors declare that the research was conducted in the absence of any commercial or financial relationships that could be construed as a potential conflict of interest.
