# Supplementary material for: TGFβ Impairs HNF1α Functional Activity in Epithelial-to-Mesenchymal Transition Interfering With the Recruitment of CBP/p300 Acetyltransferases
Source: Front Pharmacol. 2019 Aug 30;10:942. doi: 10.3389/fphar.2019.00942 (PMC6728925; doi:10.3389/fphar.2019.00942)
Supplement: Supplementary file 1 [file DataSheet_1.pdf]

Supplementary Information

Supplementary Figure Legend

Supplementary Figure S1. (A) Analysis of constitutively expressed HNF1α activity in Ras-transformed hepatocytes following TGFβ treatment. RT-qPCR analysis for the indicated genes in Ras-transformed hepatocytes transfected with pLCPX-HNF1α^{Myc} (HNF1) or the empty vector (CTR), treated with 5ng/ml TGFβ or left untreated (NT). qPCR data, obtained in triplicate and normalized to the housekeeping gene RPL34, are expressed as relative gene expression. Mean ± S.E.M of three independent experiments is shown. Statistically significant differences are reported (*p-value <0.05; **p-value <0.01; ns=not significant). **(B)** Western Blot analysis of HNF1α protein in cell extracts from one of the experiments shown in (A). CDK4 was used as loading control.

Supplementary Table 1. List of mouse primers used for RT-qPCR experiments.

Gene	Forward primer	Reverse primer
HNF4α	5'-TCTTCTTTGATCCAGATGCC-3'	5'-GGTCGTTGATGTAATCCTCC-3'
HNF1α	5'-TATCATGGCCTCGCTACCTG-3'	5' -ACTCCCCATGCTGTTGATGA-3'
TTR	5'-CCATGAATTCGCGGATGTGG-3'	5'-TCAATTCTGGGGGTTGCTGA-3'
Albumin	5'-TTCCTGGGCACGTTCTTGTA-3'	5'-GCAGCACTTTTCCAGAGTGG-3'
18S	5'-ACGACCCATTCGAACGTCTG-3'	5' -GCACGGCGACTACCATCG-3'
RPL34	5'-GGAGCCCCATCCAGACTC-3'	5' -CGCTGGATATGGCTTTCCTA -3'

Supplementary Table 2. List of mouse primers used for qPCR in ChIP experiments.

Promoter	Forward primer	Reverse primer
Albumin	5'-AGGAACCAATGAAATGCGAGG-3'	5'-AGACGAAGAGGAGGAGGAGA-3'
HNF4α	5'-ACTTGGGCTCCATAGCAAGA-3'	5'- CAGGACAGGCACAGACAAGA-3'
Neurog1	5'-CCTCCCGCGAGCATAAATTA-3'	5'-GCGATCAGATCAGCTCCTGT -3'
RPL30	5'-TAAGGCAGGAAGATGGTGG -3'	5'- CAGTGTGCTCAAATCTATCC-3'
