# Supplementary figures and images for: TGFβ Impairs HNF1α Functional Activity in Epithelial-to-Mesenchymal Transition Interfering With the Recruitment of CBP/p300 Acetyltransferases
Source: Front Pharmacol. 2019 Aug 30;10:942. doi: 10.3389/fphar.2019.00942 (PMC6728925; doi:10.3389/fphar.2019.00942)

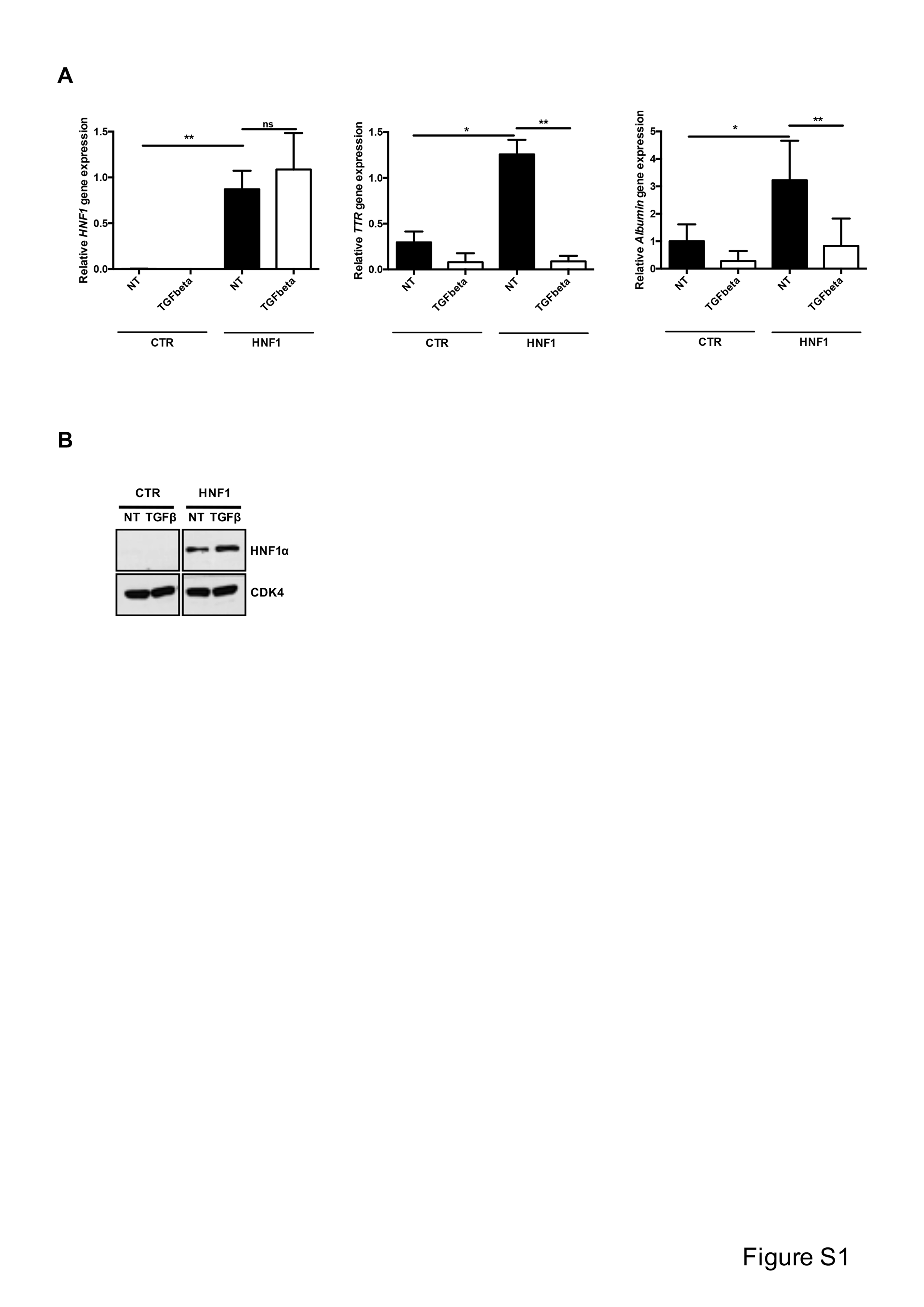

Supplement: Supplementary file 2 [file Image_1.tif]
